# Xanthogranulomatous and Emphysematous Pyelonephritis: Two Rare Entities Occurring in One Kidney

**DOI:** 10.5334/jbsr.3424

**Published:** 2024-02-14

**Authors:** Vincent Alberts, Felix Delbare, Benjamin Leenknegt

**Affiliations:** 1 Ziekenhuis Oost-Limburg, Genk, Belgium; 2 AZ Sint-Lucas, Ghent, Belgium; 3 AZ Sint-Lucas, Ghent, Belgium

**Keywords:** Xanthogranulomatous Pyelonephritis, XGP, Emphysematous Pyelonephritis, EPN, CT

## Abstract

*Teaching point:* Both xanthogranulomatous and emphysematous pyelonephritis are severe renal inflammatory disorders, occurring simultaneously in extremely rare cases.

## Case History

A 68-year-old woman presented to the emergency department with dyspnea and cognitive impairment. Her medical history included type II diabetes mellitus and severe chronic obstructive pulmonary disease (GOLD 3). Laboratory tests revealed elevated inflammatory parameters (CRP 187 mg/L). On a chest X-ray, pneumonia in the right lower lobe was diagnosed. The patient was admitted to the hospital, and antibiotic treatment was initiated. Initially, there was a clinical and biochemical improvement. However, due to worsening inflammatory parameters, liver function tests, and a high urine protein level, an abdominal CT-scan was performed.

## Image

**Figure 1:**
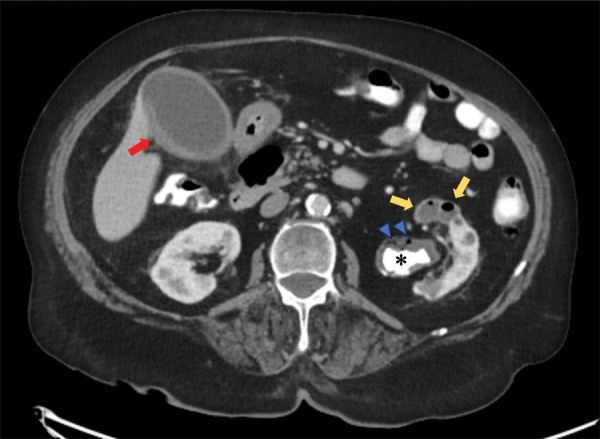
CT abdomen - axial plane.

**Figure 2:**
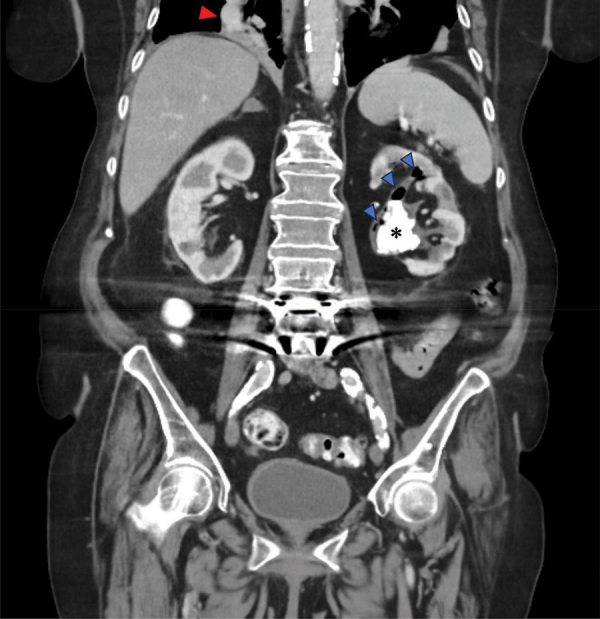
CT abdomen - coronal plane.

CT displayed a staghorn calculus (Figures 1 and 2, *) in the largely atrophic left kidney. There was a mild dilatation of the renal pelvis and calyces with wall thickening and multiple rounded fluid density cavities in the parenchyma. Gas bubbles were present in the renal pelvis surrounding the staghorn calculus (Figures 1 and 2, blue arrowheads). Some of the collections in the renal parenchyma contained gas-fluid levels (Figure 1, yellow arrows). A candidiasis infection (Candida glabrata >100 000 KVE/ml) was also identified on the urine culture test.

In addition, there was a diffusely thickened and hyperemic wall of the gallbladder, consistent with acute cholecystitis (Figure 1, red arrow). The right lower lobe pneumonia was depicted on the coronal view (Figure 2, red arrowhead).

## Comments

A very rare case is presented of combined xanthogranulomatous pyelonephritis (XGP) and emphysematous pyelonephritis (EPN), aggravated by an opportunistic infection due to antibiotic administration and weakening of the general condition. There are only a few reports of simultaneous occurrences. Even when occurring separately, XGP and EPN are severe infections of the excretory system and renal parenchyma.

XGP is a rare variant of chronic pyelonephritis caused by obstructive uropathy and infection. The urinary obstruction is almost always caused by lithiasis, commonly a staghorn calculus. *Proteus mirabilis* and *Escherichia coli* are most often involved. The combination of infection and obstruction leads to destruction of the renal parenchyma and perirenal tissue, with typical replacement by granulomatous tissue containing foamy, lipid-laden macrophages (xanthoma cells) [1]. On CT, this corresponds to the characteristically inflammatory parenchymal spaces surrounded by a thin rim of inflamed renal parenchyma (‘bear paw’ sign). Perirenal extension with fistulation to neighboring organs and structures (as the abdominal wall) may occur, and in some patients, a cutaneous abscess or fistula may be the presenting symptom. Nephrectomy is the treatment of choice.

EPN is an acute necrotizing infection of the kidney, characterized by the presence of gas in the excretory tract, the renal parenchyma, and even the perinephric tissue. The most common primary pathogen is *Escherichia coli.* It is among the most severe types of renal infection and is reported to be associated with high mortality rates [1].

Both XGP and EPN are more frequent in women and are strongly related to diabetes or immunosuppressive treatment. Almost 60% to 70% of cases are associated with uncontrolled diabetes mellitus. Contrast-enhanced CT is considered the most appropriate imaging modality for the diagnosis and assessment of the extent of the inflammatory process. A prompt diagnosis on a CT of the abdomen is key to therapeutic management.
